# Author Correction: Compact and wideband nanoacoustic pass-band filters for future 5G and 6G cellular radios

**DOI:** 10.1038/s41467-024-46362-0

**Published:** 2024-03-01

**Authors:** Gabriel Giribaldi, Luca Colombo, Pietro Simeoni, Matteo Rinaldi

**Affiliations:** NanoSI Institute, Northeastern University, Boston, MA USA

**Keywords:** Electrical and electronic engineering, Electronic devices, Developing world, Electronic devices

Correction to: *Nature Communications* 10.1038/s41467-023-44038-9, published online 05 January 2024

The Authors would like to update the Acknowledgements section. They would like to replace the Contract “HR001121S0031” with “HR0011-22-C-0088”.

